# Dual Nature of Large and Anisotropic Glass-Forming Molecules in Terms of Debye–Stokes–Einstein Relation Revealed

**DOI:** 10.1021/acs.jpcb.4c04757

**Published:** 2024-12-03

**Authors:** Abin Raj, Marzena Rams-Baron, Kajetan Koperwas, Żaneta Wojnarowska, Marian Paluch

**Affiliations:** August Chełkowski Institute of Physics, University of Silesia, 75 Pulku Piechoty 1, 41-500 Chorzow, Poland

## Abstract

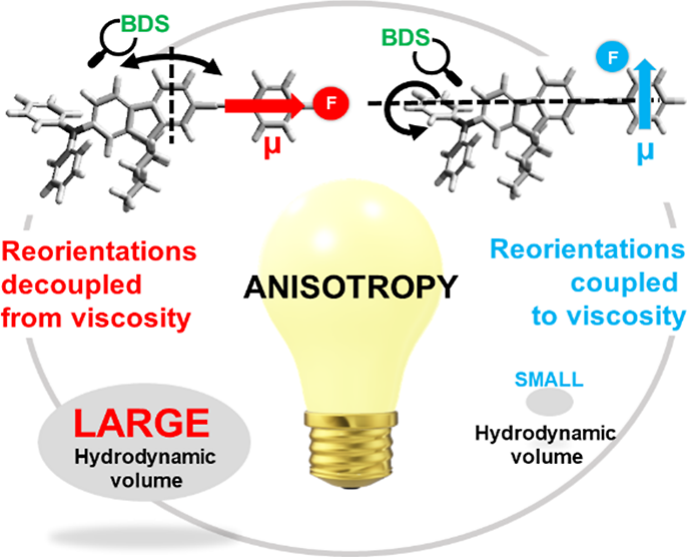

The fundamental Debye–Stokes–Einstein (DSE) relation between rotational relaxation times and shear viscosity attracts longstanding research interest as one of the most important characteristics of many glass-forming liquids. Here, we provide strong evidence, missing so far, for the relevance of anisotropy for DSE-related behavior. Dielectric spectroscopy and shear viscosity measurements were employed to get insight into the decoupling between reorientation relaxation times and viscosity for anisotropic glass-formers with dipole moments oriented parallel or perpendicular to the long molecular axis. We found that in the case of large and anisotropic molecules, the breakdown of DSE relation depends on the component of anisotropic rotation contributing to the dielectric response. Specifically, for glass-formers with dipole moment perpendicular to the long molecular axis, the DSE relation was found to be valid throughout the supercooled regime. Contrary, a departure from the DSE predictions in the intermediate supercooled regime, was observed for glass-formers where only short-axes contributions were relevant in dielectric response. MD simulations revealed differences in the mechanism of short and long axes reorientations suggesting that for anisotropic objects, not the reorientation mechanism itself, but the aspect of anisotropic motion, is the key to understanding the behavior of these systems in the context of DSE relation.

## Introduction

The transformation of a liquid into a glassy state due to a decrease in the temperature is reflected in liquid viscosity growth by 10 to 12 orders of magnitude.^1^ This enormous increase in viscosity, in turn, is accompanied by a drastic slowdown in molecular mobility.^2,3^ Although the term—molecular mobility refers to various molecular motions most important ones involve entire molecule translational and rotational motions because they are responsible for the glass transition.^4^ The key physical quantities characterizing these two types of motions are translational diffusion coefficient (*D*) and rotational diffusion correlation time (τ).

As a basis for discussing the relationship between quoted quantities and viscosity, the two fundamental laws, Stokes–Einstein (SE)^5^ and Debye–Stokes–Einstein (DSE)^6^ are routinely applied.
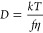
1
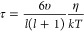
2where *f* is proportional to the size and *v* to the volume of the molecule moving in a fluid of viscosity η and *l* denotes the order of spherical harmonic which describes the reorientation relaxation process. However, it should be noted that both the SE and SED equations have some limitations since they were derived for “Brownian” particles that are large compared to solvent molecules. Despite this limitation, both were also thoroughly tested in the case of the motion of molecules surrounded by other molecules of similar or smaller size. They reasonably coincide with the experimental results down to the molecular length scale only for liquids with low viscosities, typically for temperature ranges above *T*_m_.^7^ On the other hand, the enhancement of translational diffusion with respect to viscosity is commonly observed in deeply supercooled liquids indicating the breakdown of SE relationship.^8,9^ It means that the ratio *D*η/*T* is no longer constant but instead rapidly increases as the liquid–glass transition is approached. The value of the ratio *D*η/*T* at the glass transition temperature, *T*_g_, compared to the one determined at temperatures far away from *T*_g_, usually exceeds 2 orders of magnitude. However, it was also observed that the magnitude of the decoupling is systematically reduced when the size of the molecular probe increases.^8^ All these observations were interpreted through the effect of spatially heterogeneous dynamics.^10^ This view, however, was contested partially in ref (11).

The situation is somehow different when considering experimental results for rotation. The ratio η/τ*T* depends much less on temperature than *D*η/*T* indicating only a slight deviation from DSE relation as the temperature is lowered to near *T*_g_.^12−14^ Richert et al. compared dielectric relaxation data and viscosities for several glass-formers (e.g., salol, trinaphthyl-benzene, dibutyl phthalate, α-phenyl-*o*-cresol) indicating that decoupling appears to be absent for temperatures above crossover temperature while in more viscous regime the dielectric relaxation becomes a factor of 2 to 5 faster relative to viscosity.^15^ Thus, the authors confirm dielectric relaxation enhancement compared to viscosity reported before by Chang and Sillescu.^16^ On the other hand, for a few low molecular glass-forming liquids, there is evidence of no decoupling of the viscosity from the dielectric relaxation times.^17,18^ Besides, the DSE relation was tested using the experimental data obtained from pressure-dependent measurements, proving its validity.^19^ Due to the lack of decoupling phenomena in the mentioned examples, it cannot definitively be combined with the spatial heterogeneity inherent to supercooled liquids. Besides, as argued by Chang and Sillescu in ref (16) heterogeneity cannot explain why dielectric relaxation times decouple from viscosity as both are local processes. However, the presence or absence of decoupling between viscosity and dielectric relaxation times was rationalized based on the idea that different dynamic variables experience different degrees of intermolecular cooperativity by considering the temperature evolution of the relaxation spectrum.^13,20^

The effect of the size of the probe molecule was studied not only on the translational molecular motion, as already mentioned, but also on molecular rotation. In this context, results obtained by Ediger and co-workers and reported in ref (14) deserve to be mentioned. The authors pointed out that the width of the relaxation spectrum determined from time-resolved optical spectroscopy measurements systematically decreased with increasing the probe size. Thus, an essential aspect of the study of molecular rotation becomes also the analysis of the spectral shape of the corresponding response function. Interestingly, although, aromatic probes in OTP hosts investigated previously were anisotropic molecules, the possible role of anisotropy for DSE-related behavior was excluded.^14^ Large molecules are generally expected to follow hydrodynamic behavior as predicted by the DSE relation, however, deviations may occur if the large molecule is also anisotropic. Since a molecule with a nonspherical shape can exhibit different relaxation rates around different molecular axes, deviations from the DSE-predicted behavior may or may not occur depending on which aspect of the motion we are investigating. Thus, for large and anisotropic objects, a more complex picture regarding DSE compliance should be expected.^21^

Here, we present a complete study of DSE relation validity for large, partially rigid, and anisotropic molecules. We present strong evidence for the importance of anisotropy for DSE-related behavior, overlooked in the previous studies.^14^ The main focus of this work is testing the DSE equation in the case of anisotropic rotation probed by dielectric relaxation spectroscopy. More specifically, we studied the rotational motion of three similar anisotropic rigid and sizable molecules, which only differ in the orientation of the dipole moment. For one sample, the dipole moment is perpendicular to the long main molecular axis, while in two other samples, the dipole moment is parallel to this axis.

**Figure 1:**
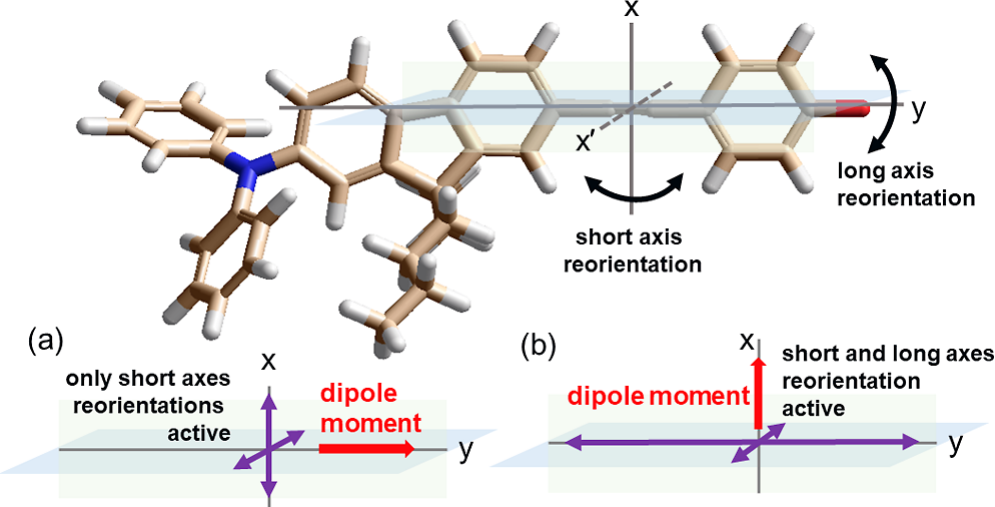
Different contributions to the dielectric response depending on the alignment of the dipole moment vector, μ, with respect to the long molecular axes of an anisotropic sizable molecule. The case when μ is parallel (a) or perpendicular (b) to the long molecular axis.

Broadband dielectric spectroscopy provides information about the process of molecular reorientation through the dipole–dipole correlation function. Thus, the alignment of the dipole moment with respect to the molecular axis of a rigid anisotropic molecule plays a crucial role here. In a particular case, if the dipole moment is parallel to one of three main molecular axes, the rotation around this axis will not be active in dielectric response because the dipole moment’s orientation changes do not occur. Instead, the fluctuation of the dipole moment brought about by molecules’ orientations around the two remaining main molecular axes will constitute the dielectric response. Recalling the samples examined in this work, one can distinguish two cases, i.e., when the total dipole moment is parallel (M-para-F, M-2F) or perpendicular (M-ortho-F) to the long molecular axis. In the first group, the dielectric response will only reflect the reorientations around short axes. In contrast, in the second one, reorientation around the long and short axes must be considered. As a result, we experimentally observed the dielectric response determined by two different but interdependent contributions as schematically illustrated in Figure 1.

## Methods

To gain insight into the effect of anisotropy on molecular dynamics in terms of DSE relation we compared dielectric relaxation times, τ_BDS_, and viscosities, η, measured over a broad range of temperatures (where both quantities changed by 10 or 12 orders of magnitude, respectively), for three anisotropic molecules whose rotation about the short and long axes differed significantly. Sizable molecules varying only in the position of the fluorine atom(s) and therefore dipole moment orientation (see Figures 3 and S1 for the results of the dipole moment calculations), referred to as M-2F, M-para-F, and M-ortho-F, were synthesized on request by Trimen Chemicals (Łódź, Poland). Their dielectric spectra in a frequency range from 10^–3^ to 10^7^ Hz, were obtained using a Novo-Control GMBH Alpha dielectric spectrometer with the Novocool temperature-control system, using stainless steel electrodes (diameter = 15 mm, distance = 0.1 mm). The shear viscosity measurements were carried out using an ARES G2 rheometer using aluminum parallel plates of diameter 4 mm. The rheological experiments were performed with a strain equal to 0.01% in the vicinity of the liquid glass transition, *T*_g_; later the strain was increased by 1 order of magnitude with every 10 K.

## Results & Discussion

**Figure 2:**
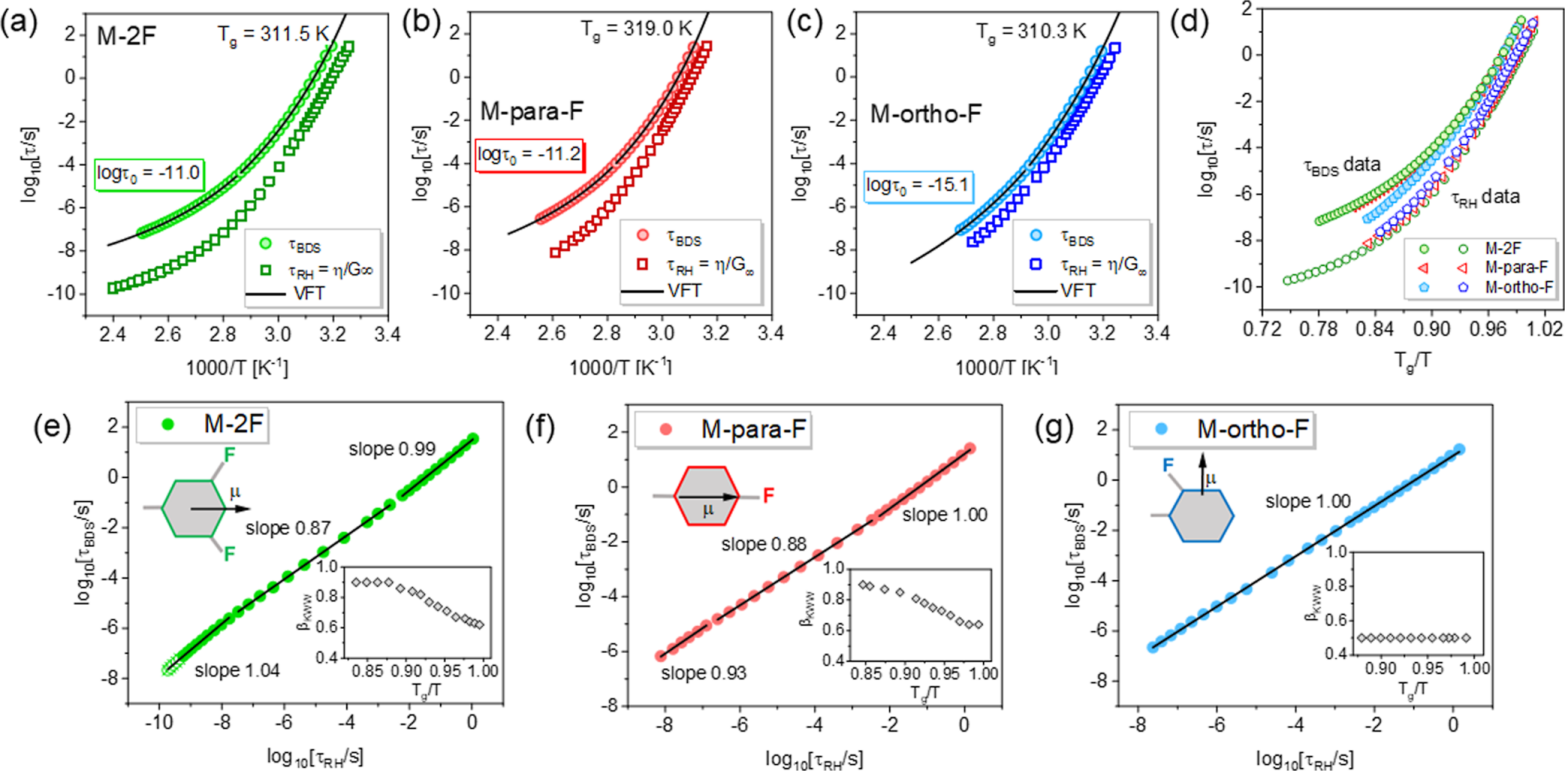
Comparison of the temperature dependence of dielectric τ_BDS_, and mechanical τ_RH_, relaxation times for M-2F (a), M-para-F (b), and M-ortho-F (c). (d) Relaxation map presenting τ_BDS_ and τ_RH_ data depicted as *T*_g_/*T* for all anisotropic molecules. The lower panels (e–g) compare the mechanical and dielectric relaxation times in the same temperature frame in terms of decoupling phenomena. Insets demonstrate how the value of the β_KWW_ parameter depends on *T*_g_/*T*.

The temperature dependence of dielectric relaxation times, τ_BDS_, determined from the dielectric permittivity spectra, ε*(*f*) = ε′(*f*) + *i*ε″(*f*) by using Havriliak–Negami parameters^22^ are shown in Figure 2a–c. To parametrize the τ_BDS_(*T*) data we had to use two Vogel–Fulcher–Tamman functions (VFT), τ_BDS_(*T*) = τ_0_ exp[*DT*_0_/(*T* – *T*_0_)],^23−25^ separately for low- and high-temperature regimes as confirmed by Stickel’s analysis,^26^ see Figure S2 and Table S1 for the corresponding values of VFT fitting parameters i.e. *D*, *T*_0_, and log τ_0_. Results presented in Figure 2a–d clearly demonstrated that the reorientation dynamics of M-2F, M-para-F, and M-ortho-F probed dielectrically varied substantially. Observed dielectric behavior reflects the component of anisotropic rotation contributing to the dielectric response and carried some signatures of long or short-axis reorientations whose relevance in particular samples varied. The most striking difference is the discrepancy between the high-temperature limit of τ_BSD_(*T*^–1^) dependencies between M-ortho-F and others. For M-ortho-F, the high-temperature limit corresponds to shorter relaxation times. It is manifested by a smaller value of the pre-exponential factor τ_0_ (log τ_0_ = −11.0 for M-2F, log τ_0_ = −11.2 for M-para-F, vs log τ_0_ ≈ −15.1 for M-ortho-F). As τ_0_ ∼ (2π*I*/*k*_B_*T*)^0.5^ (*I* is the moment of inertia, *k*_B_ is a Boltzmann constant, *T* is temperature).^27^ The observed difference is due to a smaller moment of inertia *I* characterizing the long-axes reorientations contributing to the dielectric response of M-ortho-F.^28^ For such motion, the mass is distributed closer to the axis of rotation compared to the reorientation about the short axis relevant in M-para-F and M-2F.

**Figure 3:**
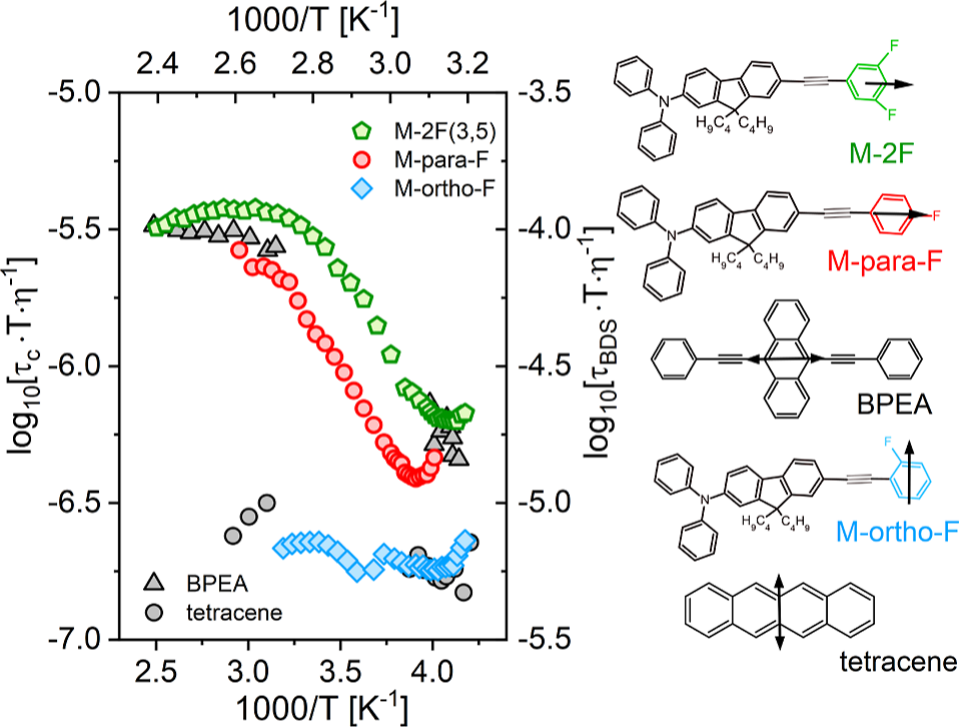
Critical analysis of the departure from the DSE-predicted behavior based on temperature evolution of τ·*T*·η^–1^ product. Data for BPEA and tetracene were taken from ref (14) where rotational correlation times τ_c_ were determined from time-resolved optical spectroscopy. Notice the consistency in the pattern of temperature evolution of τ·*T*·η^–1^ ratio with the anisotropic rotation components probed experimentally and determined by the alignment of μ (one-side arrows) or absorption transition dipoles relevant for BPEA and tetracene (depicted as two-way arrows) with respect to the longer molecular axes of a given system. Adapted from ref (14) with the permission of AIP Publishing.

Along with the dielectric data, the temperature dependence of mechanical relaxation times calculated from viscosity data (see Figure S3) using the Maxwell relation τ_RH_ = η/*G*_∞_^17^ are depicted in Figure 2a–c. In calculations, we used the following value of the infinite-frequency shear modulus *G*_∞_ determined from *G*′(ω) spectra *G*_∞_ = 4.7 × 10^8^ Pa (for M-2F), *G*_∞_ = 4.0 × 10^8^ Pa (M-para-F), and *G*_∞_ = 5.0 × 10^8^ Pa (M-ortho-F). The visual comparison of the τ_BDS_ (*T*^–1^) and τ_RH_ (*T*^–1^) data shows another difference among the investigated materials. For M-ortho-F, the τ_RH_ and τ_BDS_ had exactly the same character of *T*^–1^ dependence indicating coupling between mechanical and dielectric relaxation times. Distinct behavior was observed for M-para-F and M-2F. A more precise insight was provided by analyzing a double logarithmic plot of log τ_RH_ versus log τ_BDS_ comparing both quantities within the same temperature frames, see Figure 2e–g. An apparent proportionality (i.e., slope equal to unity) between τ_RH_ and τ_BDS_ was observed over the entire temperature range only for M-ortho-F. The analysis for M-2F and M-para-F resulted in more intricate outcomes, as the coupling between τ_RH_ and τ_BDS_ was temperature-dependent. At sufficiently high *T* and in the vicinity of *T*_g_, the τ_RH_ and τ_BDS_ were coupled; in the intermediate *T* regime, decoupling was found. The DSE relation breaks down at *T* ≈ 1.16*T*_g_ for M-2F and 1.14*T*_g_ for M-para-F. Subsequently, the decoupling was maintained up to *T* = 1.04*T*_g_ for both samples. To analyze the correlation between viscosity and τ_BDS_ in terms of DSE relation the product of τ_BDS_·*T*·η^–1^ was depicted as a function of 1000·*T*^–1^ in Figure 3. A flat line denotes that viscosity and dielectric relaxation times follow each other as predicted by DSE relation. This is true only for M-ortho-F for which DSE was found to be valid in the entire supercooled regime. For M-2F and M-para-F a decrease of τ_BDS_·*T*·η^–1^ product on approaching *T*_g_ denoted a breakdown of the DSE relation. The trend shown in Figure 3 indicates that dielectric relaxation times on cooling become faster than those predicted by the DSE from high-temperature data.

In the past, the breakdown of DSE relation for many molecular systems was discussed in terms of loss peak broadening and attributed further to growing cooperativity on approaching liquid-to-glass transition. An explanation, given by the Coupling Model, combined the decoupling phenomena with various degrees of intermolecular cooperativity.^20^ The insets in Figure 2e–g show how the width of the dielectric loss spectra changed with temperature for each sample. The broadening of ε″(*f*) spectra was quantitatively described by the β_KWW_ parameter (0 > β_KWW_ ≥ 1), an exponent of the Kohlrausch–Williams–Watts (KWW) function,^29,30^ φ(*t*) = exp[−(*t*/τ_BDS_)^β_KWW_^, see Figure S4. For M-2F and M-para-F the spectral shape evolved with temperature. The broadening of loss peak on cooling was expressed by a decreasing value of the β_KWW_ parameter, see insets in Figure 2e,f. Interestingly, near *T*_g_ and at high *T*, the temperature sensitivity of β_KWW_ becomes weaker. In contrast, for M-ortho-F (Figure 2g) the collected ε″(*f*) spectra could be satisfactorily described by the same value of β_KWW_ = 0.5 over the entire temperature range. The temperature-independent distribution of relaxation times for M-ortho-F was also confirmed by the analysis of ε″(*f*) spectra employing the Havriliak–Negami function^31^ (see Figure S5). It means that the presence of decoupling in investigated molecules was correlated with the sensitivity of the spectrum shape to temperature changes, in accordance with previous reports dealing with the decoupling phenomenon in small isotropic molecules.^19,20,32^

Surprisingly, the results presented herein are consistent with Ediger data^14^ in terms of a relation between the value of the β_KWW_ parameter and probe size. Specifically, the authors found that probes larger in terms of hydrodynamic volume show significantly larger values of the β_KWW_ parameter, which was also true in the case of sizable molecules investigated herein. The environment experienced by a large molecule is averaged differently than in the case of a small object making the immediate molecular environment, probed across larger length scales, more uniform. Paeng et al.^33^ reached similar conclusions, investigating how systematic changes in the length of flexible linkers and the number of tethering points affect the probing of glass transition dynamics in the host polymer. The authors found that the mechanism of how probes report on the dynamics of the host polymer is influenced by probe size. Smaller probes captured the full spectrum of polymer dynamics, while larger probes during averaging tended to overlook the faster components of dynamic heterogeneity, leading to a higher β_KWW_ value. In our study, despite similar masses and shapes, the effective volume required to implement the long or short-axis reorientation for sizable molecules will vary considerably. It is reflected by values of the hydrodynamic volume calculated from the DSE relation for studied systems which were significantly higher for M-para-F and M-2F than that obtained for M-ortho-F. At *T* = 1.17*T*_g_*V* = 909 cm^3^/mol for M-2F, *V* = 609 cm^3^/mol for M-para-F, and *V* = 60 cm^3^/mol for M-ortho-F. One must realize that for a nonspherical object, the hydrodynamic volume calculated from the DSE relation is an abstract concept, defined as the volume of an imaginary spherical particle that shares the same relaxation time and viscosity at a specific temperature as the molecule studied. Consequently, depending on whether we analyze short or long axes rearrangements the corresponding hydrodynamic volume calculated from DSE can be small or large regardless of the actual geometric size of the object under study. In this context, anisotropic molecules are unique because, despite the identical size, they can virtually exhibit dynamic behavior typical for small or large objects (e.g., in terms of the relationship between the effective volume and the value of the β_KWW_ parameter) depending on the aspect of motion being studied. In light of the above, the M-ortho-F behaves as a small object that experiences the environment differently than M-para-F and M-2F, leading to a lower β_KWW_ value.

The comparison with data from ref (14) is continued in Figure 3 where we recall the previously published results for tetracene and BPEA. These data were taken from a study on fluorescent probe molecules embedded in a small OTP host. The probes were similar in size to OTP or larger than the host, and their anisotropic shape differed from the spherical shape of the host. Thus, basically, our research is different as we study neat supercooled liquids with molecules of uniform size and the same anisotropy. Nevertheless, the comparison in Figure 3 shows interesting parallels. BPEA, the large probe that was found to rotate with almost a single exponential correlation function, behaves similarly to M-2F and M-para-F. Tetracene, a smaller size probe with a greater distribution of relaxation times behaved similarly to M-ortho-F. Surprisingly, our results not only satisfied the previously observed relationship between the hydrodynamic volume and the β_KWW_ parameter but also showed a similar pattern of behavior regarding the degree of decoupling. The ratio of *R* = [τ_BDS_·*T*·η^–1^]_*T*_g__·[τ_BDS_·*T*·η^–1^]^−1^_high-*T*_ was used to compare a magnitude of deviation from DSE behavior for materials depicted in Figure 3. For BPEA *R* = 6 while for tetracene *R* = 2.^14^ Accordingly, *R* = 5.5 for M-2F, *R* = 5 for M-para-F and *R* = 1 for ortho-F. Although the authors ignore this effect, indicating rather small deviations from DSE for all probes, it is clear that the behavior reported for BPEA and tetracene was consistent with those observed for sizable systems studied in our work. What these molecules have in common is rotational anisotropy and the different relevance of short and long-axis components to the overall response which in the case of samples studied herein was determined by the dipole arrangement, also depicted in Figure 3.

Although the behavior of the systems studied in this work is consistent with that previously reported by Ediger, no one has yet linked these effects with anisotropy. Even, the authors denied its possible impact, thus, our results point out a new perspective on this fundamental problem.^14^ The materials studied herein are ideal for this purpose as discussed parameters, τ_BDS_, β_KWW_, or *R* (decoupling degree), can be undeniably related to the types of anisotropic reorientations involved. One can note that for investigated systems compliance or noncompliance with temperature-dependent behavior of η and τ_BDS_ suggested by DSE can be correlated with the character of motion probed by dielectric method. The deviation from DSE was found for samples whose dielectric response was governed by short axes contributions. Contrary, DSE compliance was established for the sample, M-ortho-F, where both short and long axes components constitute a dielectric response. It means that the component of anisotropic rotation probed experimentally was very closely related to DSE-related behavior underlining the overriding role of anisotropy, overlooked so far.

What is not entirely clear, is the large difference in the values of the β_KWW_ parameter between individual samples. One explanation can be offered by differences in the effective volume and different ways of environment sampling, similar to ref (14). An alternative way is to take into account an intrinsically nonexponential relaxation function originating from distinct rotation rates about different molecular axes of an anisotropic system. More precisely, the narrow distribution of relaxation times observed for M-2F and M-para-F can be attributed to the greater uniformity of short axes reorientations contributing to the dielectric response of these molecules. Since the dipole moment in M-2F and M-para-F is directed along the long axis, the long axis reorientation does not affect the dielectric response. The experimentally observed short axes reorientations are both inherently slower and equivalent in terms of mass distribution along reorientation axes. In M-ortho-F, the situation is more complex because the dielectric behavior is determined by the combination of reorientations along the short and long axes. Such motions are not independent and observed peak broadening cannot be regarded as an effect of their superposition. Their greater complexity may underlie the broader distribution of relaxation times observed for M-ortho-F in comparison to M-2F and M-para-F. Unfortunately, based on experimental data, we are not able to isolate the particular spectral contributions (only from the long axis or only from one of the short axes), thus, such a conclusion cannot be undeniably drawn. In addition, in M-ortho-F the fluorophenylene unit has a substantial rotational freedom as the molecule belongs to the group of molecular rotators.^34,35^ The internal rotation of the fluorophenylene unit in M-ortho-F contributes to the dielectric response as a fast β-process.^35^ Since the time scales of the α and β processes are well separated, the β process is not expected to affect the time scale of molecular reorientation. The internal dipole rotation is so rapid that, from the point of view of the much slower reorientation of the molecules, the change in the position of the dipole moment associated with the internal mobility is averaged out. Nevertheless, additional internal mobility may contribute to the larger distribution of relaxation times τ_BDS_ observed for M-ortho-F.

To get better molecular insight into the mechanism of the molecular reorientations around the short and long axes, we performed MD simulations for highly asymmetric model molecules. Their scheme and the description of the simulation procedure are presented in the Supporting Information. We define two vectors, i.e., the first one is placed alongside the molecular core and links two atoms of its first rhombus-like element. It corresponds to the dipole moment vector oriented alongside the longest molecular axis (we denote it as *v*_∥_). The second vector connects atoms placed alongside the shorter diagonal of the rhomb and corresponds to the dipole moment vector oriented perpendicularly to the longest molecular axis (*v*_⊥_). The rotational dynamics of the first vector involve only short-axis reorientations while for the second vector, both the short and the long axes reorientations are relevant, see Figure 1. The information considering the character of the reorientations of particular vectors can be deduced from the ratio , where ⟨τ⟩_s_ is the average relaxation time of the self-correlation function ( is an average relaxation time) probed by the respective Legendre polynomial, i.e., the first *P*_1_(*x*) = *x* and second *P*_2_(*x*) = (3*x*^2^ – 1)/2, and *x* is a cosine of the rotation angle of the studied vector made during a given time interval.^36^ If the ratio is close to unity, the molecular rotation tends to occur through substantial jumps, bringing the molecule through the potential energy minima.^37^ Alternatively, the reorientation can be realized by the progressive steps of the small jumps, as assumed by the standard rotational diffusion model.^38^ Then  value is expected to be equal to 3. In the Supporting Information we show the detailed description of performed analysis. Its main outcome is that  ratio calculated for *v*_∥_ and *v*_⊥_ differs significantly. Namely, for *v*_∥_ we get that  at *T* = 400 K and 2.73 at *T* = 160 K, while for *v*_⊥_ the value of  is smaller, i.e., 2.16 at *T* = 400 K and 2.04 at *T* = 160 K. It means that the reorientation of anisotropic glass formers with a dipole moment parallel to the long molecular axis take place mostly through a small-step diffusion process. Contrary, reorientations in molecules with a dipole moment perpendicular to the long molecular axis show a more complex behavior, characterized by a 2-fold mechanism involving also jump-type motions. These results show that in the case of anisotropic objects, not only the time scale and effective volume associated with a given reorientation motion are different, but also the reorientation mechanism itself is different, at least at the high temperatures at which MD simulations were performed. Our results can be compared with recent reports on supercooled water molecules where the calculated contribution from jump-like diffusion, which grows on cooling, quantitively describes the deviation from SE-predicted behavior observed experimentally. In ref (39) authors estimated the jump-only diffusion coefficient, which clearly decouples from viscosity. On the other hand, when the jump-only diffusion is extracted from the overall molecular diffusion, the remaining diffusion coefficient stays strongly coupled with viscosity. These conclusions were drawn for the translational motion of a system whose dynamic properties are governed by the rearrangement of the hydrogen bond network.^40^ Even more, the rotational relaxation times probed by the different Legendre polynomials are suggested to be entirely inconsistent with the rotational diffusion coefficient for this system.^41^ Nevertheless, with these results in mind, the question arises whether the reorientation mechanism is somehow related to the DSE breakdown in the studied systems. At this point, we can only refer to the high-temperature regime (relevant for MD simulations) where no discrepancies with DSE predicted behavior are found in Figure 2e–g. An attempt to interpret the experimental results together with the MD simulation results in the context of DSE agreement shows that despite the different reorientation mechanisms found in MD simulations for different aspects of the anisotropic reorientation, the relaxation times τ_G_ and τ_BDS_ remain coupled for all systems in the relevant high-*T* regime. This may suggest that for anisotropic molecules investigated herein, the reorientation mechanism (jump vs small angle) may not be the decisive factor for DSE violation/preservation. It further implies that for anisotropic objects, not the reorientation mechanism itself, but the aspect of anisotropic motion, may be the key to understanding the behavior of these systems in the context of DSE relation.

## Conclusions

In summary, our study revealed a relationship between the nature of anisotropic rotation (involving short and/or long-axis rotations) contributing to the dielectric response and compliance or noncompliance with the DSE law, emphasizing the significance of anisotropy—an aspect overlooked in previous research. We found that mobility involving the combination of short and long axes reorientations remained strongly coupled to the viscosity throughout the supercooled regime. In contrast, when only short-axis reorientations were contributing to the dielectric response, the more complex behavior and violation of the DSE law at intermediate temperatures in a supercooled regime were observed. Finally, the molecular picture of the short and long axes reorientation mechanism was given by MD simulations. Our results suggest that for sizable anisotropic glass formers, not the involved reorientation mechanism (jump vs small angle), but an aspect of anisotropic reorientation may be relevant for a full understanding of dynamical behavior in the future, also in the context of DSE relation.

## Supporting Information Available

Dipole moment calculations, supporting analysis of dielectric spectra, temperature dependence of shear viscosity data, molecular dynamics simulations details (PDF)
