# Supplementary material for: Dual Nature of Large and Anisotropic Glass-Forming Molecules in Terms of Debye–Stokes–Einstein Relation Revealed
Source: J Phys Chem B. 2024 Dec 3;128(49):12154–60. doi: 10.1021/acs.jpcb.4c04757 (PMC11647875; doi:10.1021/acs.jpcb.4c04757)
Supplement: Supplementary file 1 — jp4c04757_si_001.pdf [file jp4c04757_si_001.pdf]

Supporting Materials

Dual Nature of Large and Anisotropic Glass-Forming Molecules in Terms of Debye-Stokes-Einstein Relation Revealed

Abin Raj, Marzena Rams-Baron*, Kajetan Koperwas, Żaneta Wojnarowska, Marian Paluch

August Chełkowski Institute of Physics, University of Silesia, 75 Pulku Piechoty 1, 41-500 Chorzow, Poland

*email: marzena.rams-baron@us.edu.pl

Table S1.VFT fitting parameters

	VFT1 (low-T regime)			VFT2 (high T-regime)		
	$\log\tau_0$	D	T_0	$\log\tau_0$	D	T_0
M-2F	-14.2 ± 0.2	8.6 ± 0.3	253 ± 1	-11.0 ± 0.1	3.7 ± 0.1	281 ± 1
M-para-F	-13.4 ± 0.2	7.2 ± 0.3	265 ± 1	-11.2 ± 0.1	4.0 ± 0.1	284 ± 1
M-ortho-F	-18.1 ± 0.5	13.9 ± 1.0	239 ± 3	-15.1 ± 0.4	8.4 ± 0.9	256 ± 5

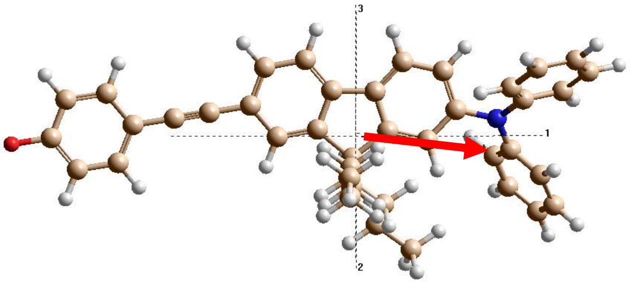

(a) M-para-F (μ= 2.2 D)

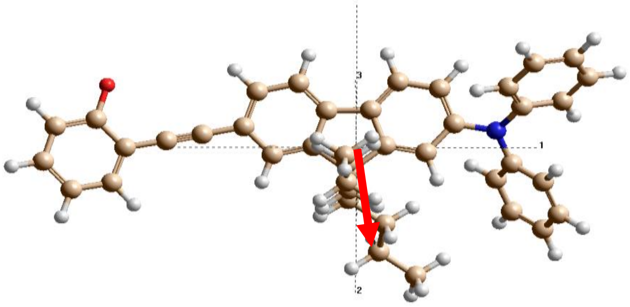

(b) M-ortho-F (μ= 1.9 D)

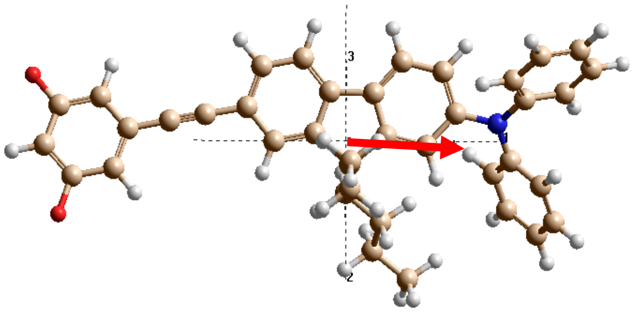

(c) M-2F (μ= 2.9 D)

Fig. S1. Chemical structure of M-para-F, M-meta-F, and M-2F. The red arrow denotes the alignment of the dipole moment vector, μ, according to the AM1 semi-empirical method calculations. Assignment of atoms: brown - carbon atoms, white - hydrogen, red - fluorine, blue - nitrogen.

(a)

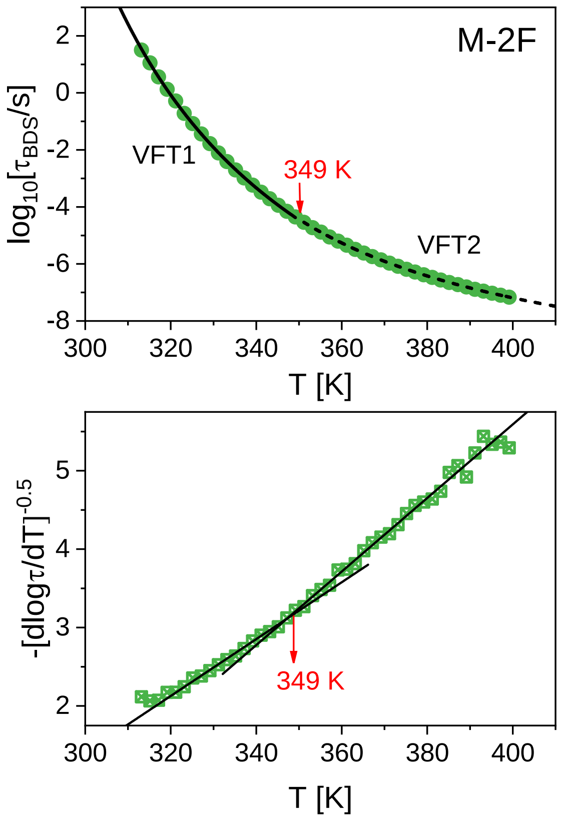

(b)

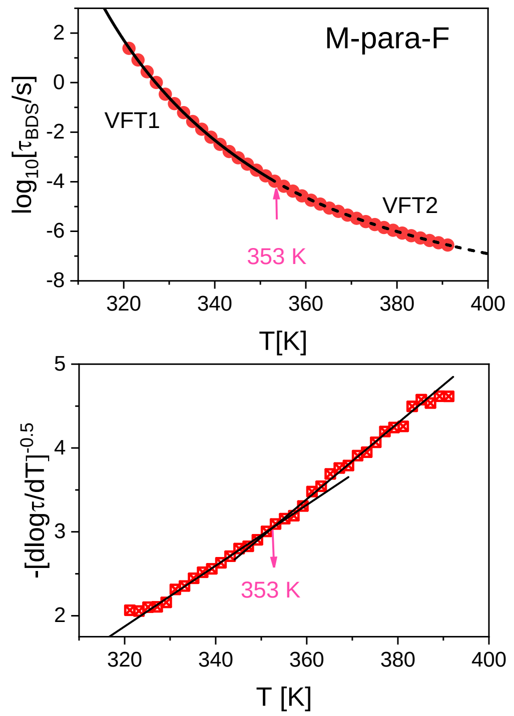

(c)

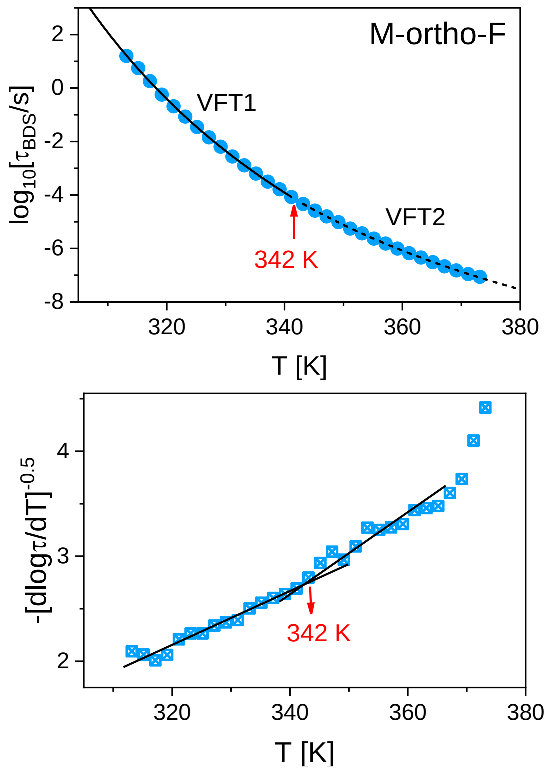

Fig. S2. Results of Stickel analysis, a derivative method of data linearization used to determine the VFT fit boundaries. The arrow indicates the so-called crossover temperature which separates regions with different VFT fitting parameters.

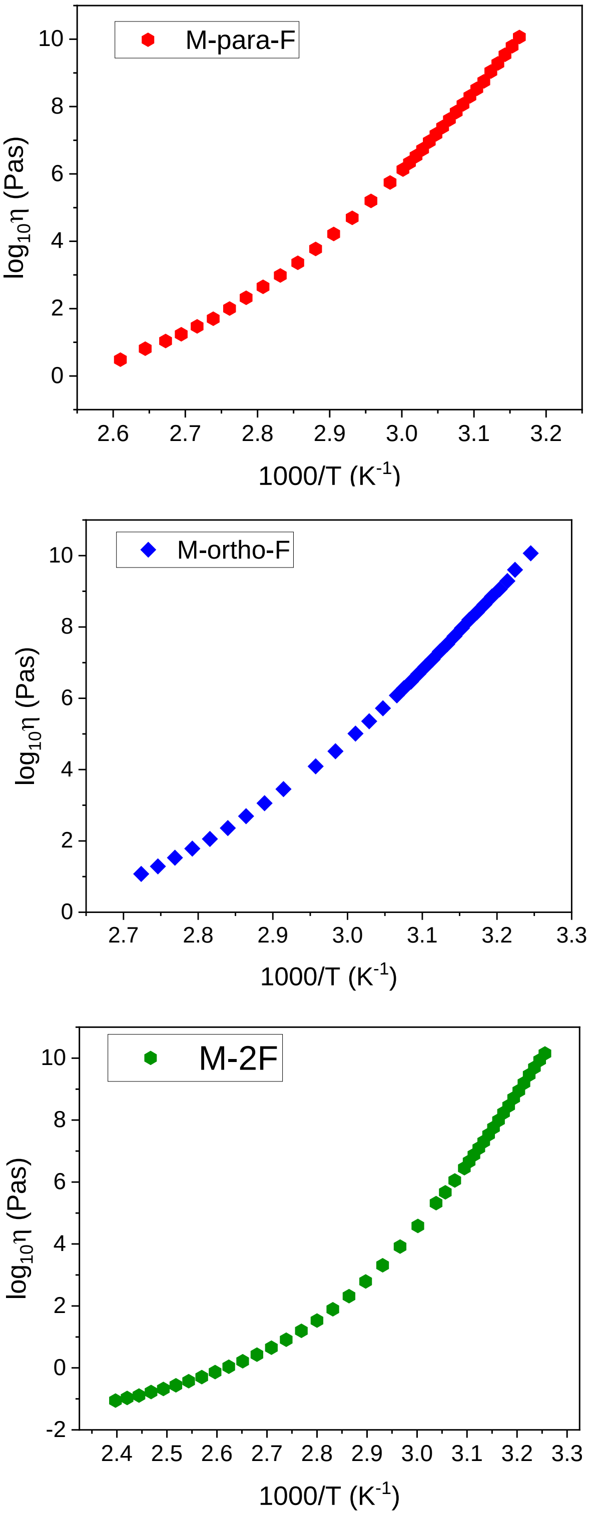

Fig. S3. Viscosity η as a function of 1000 T^{-1}.

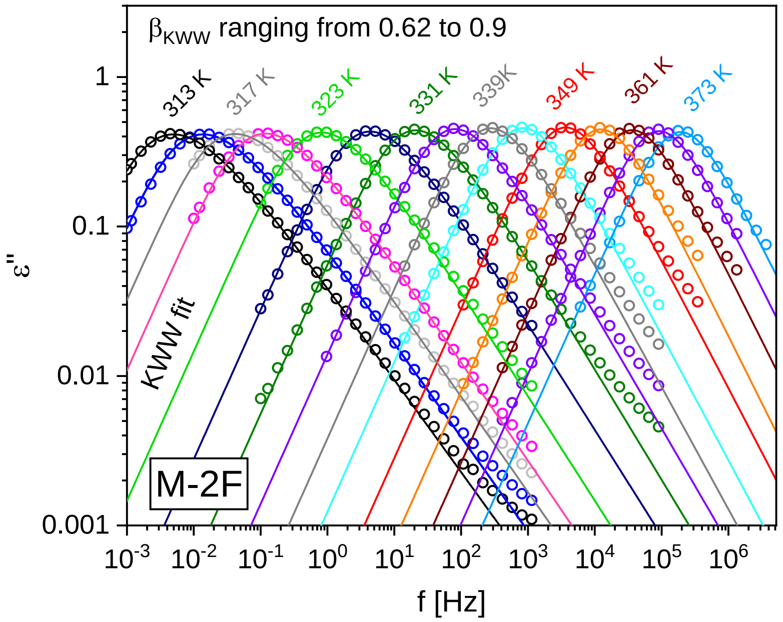
βKWW ranging from 0.62 to 0.9
313 K
317 K
323 K
331 K
339K
349 K
361 K
373 K
KWW fit
M-2F
ε''
f [Hz]

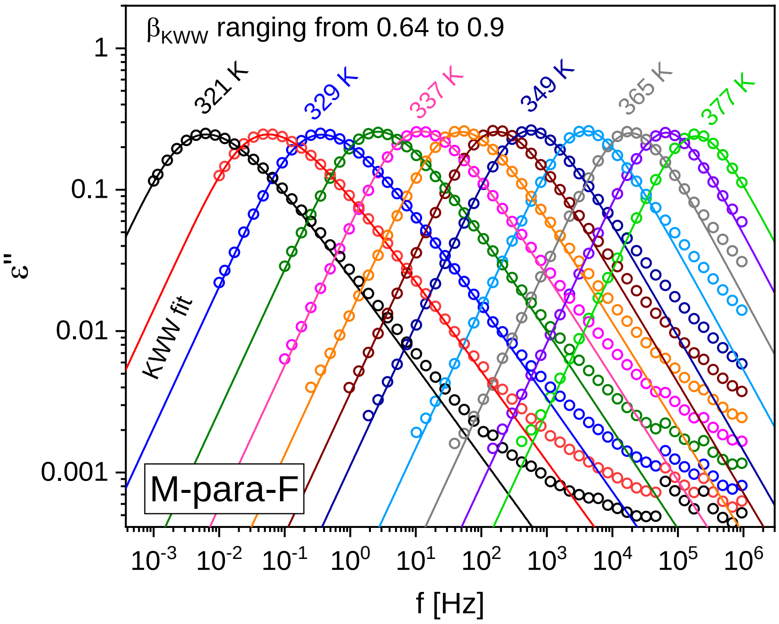
βKWW ranging from 0.64 to 0.9
321 K
329 K
337 K
349 K
365 K
377 K
KWW fit
M-para-F
ε''
f [Hz]

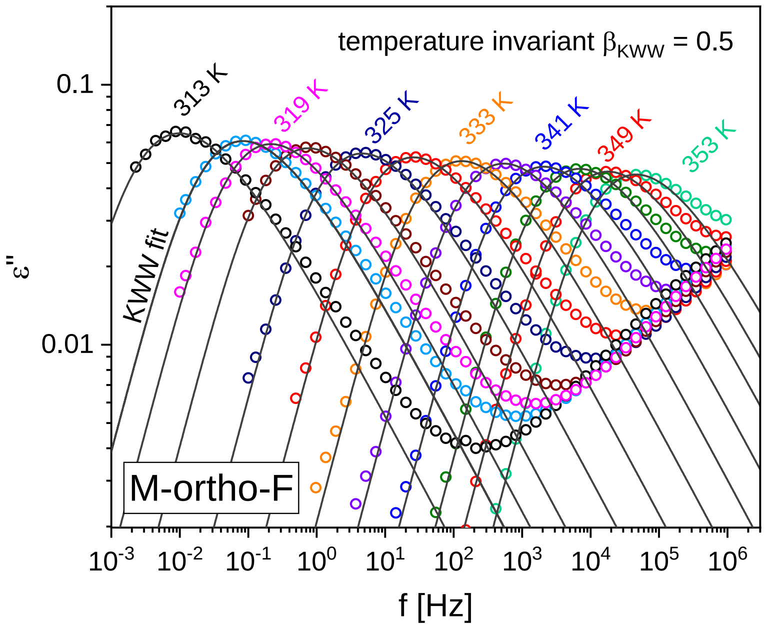

Fig. S4. The representative dielectric loss spectra for M-2F, M-para-F, and M-ortho-F. The symbols represent the dielectric loss data, while the solid lines denote KWW fitting functions. The corresponding β_{KWW} parameters for KWW fits are plotted as a function of T_g/T in Figs.2 e-f (insets).

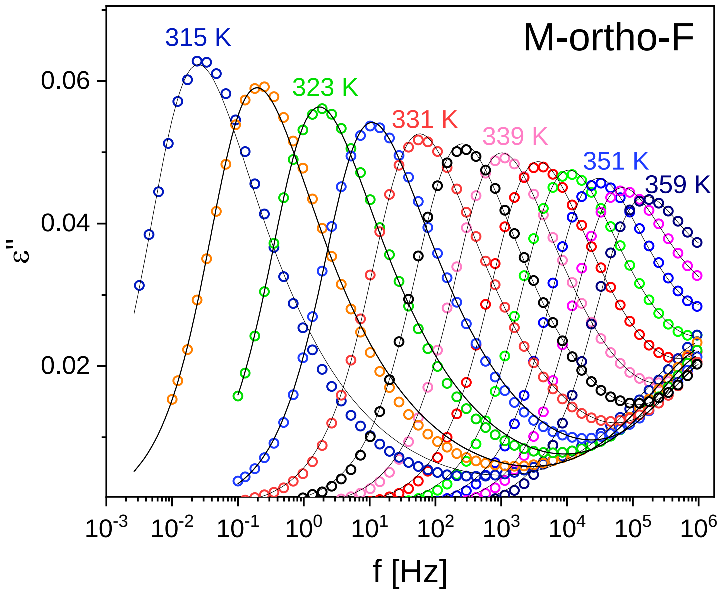

Fig. S5 The analysis of ε"(f) spectra for M-ortho-F employing the Havriliak-Negami function with temperature-invariant shape parameters $\alpha_{HN} = 0.84$ $\beta_{HN} = 0.39$.

Molecular Dynamics Simulations Details

The simulations of the molecular dynamics at constant volume (V=1650nm^3) and temperatures equal to 400K and 160K were carried out using the GROMACS software [32–36] with the Nose-Hoover thermostat [37–39]. The equations of motion were integrated using the velocity-Verlet algorithm [40] with a time step equal to $0.001ps$. The system consists 5000 molecules, which are constructed from 15 identical atoms belonging to 3 rhombus-like elements constituting the molecular core or to triatomic (freely joined) side chain, see Fig. S6(a). The mass and interaction potential parameters of atoms are defined by OPLSAA force field parameters delivered for the carbon atom of the benzene ring. [41] The only modification we made refers to atoms' charges which are set to $0.0e$. The truncation of the intermolecular interaction at a distance equal to $1.065\ nm$, which is 3 times higher than the σ parameter of the intermolecular interaction potential.

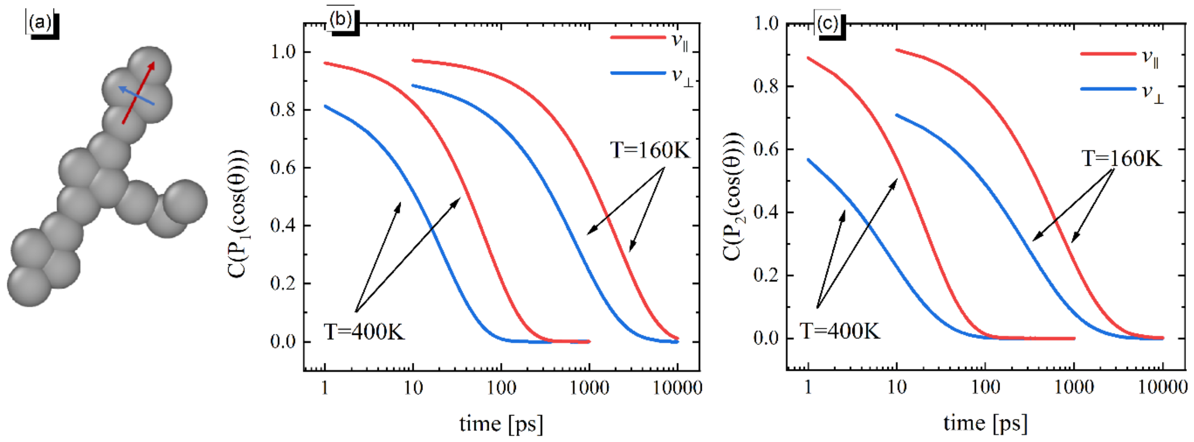

Fig. S6 The scheme of the model structure (a) and calculated correlation for the first (b) and the second (c) Legendre polynomial.

The simulations intended for data collection had been proceeded by the system equilibration which lasted 200ns and 2000ns for T=400K and 160K respectively. During these times the Incoherent Intermediate Scattering Function calculated for the maxim of the structure factor decays more than 10 times. The analysis of the time-dependent correlation functions starts from the introduction of the two vectors of our interest, i.e., $v_{\parallel}$ and $v_{\perp}$. The vectors are schematically presented in Fig.S6(a) as the red and blue arrows respectively. As can be seen, both vectors link the atoms of the first rhombus-like element, but they are differently oriented with respect to the longest molecular axis. In this way, the reorientation of the vectors activates various aspects of the molecular motions. The calculated correlation functions are shown in Fig. S6(b) and (c) for the first and the second Legendre polynomial. In both cases, θ is the angle between the studied vector at the initial position and the same vector at a later time.
